# Pyometra—An Atypical Complication Following LeFort Colpocleisis: Narrative Review

**DOI:** 10.1007/s00192-025-06335-3

**Published:** 2025-09-30

**Authors:** Emilio Chala Saad, Mariana Abril Barreto, Camilo Fonseca Guzmán, Omar Leonardo Gómez Polania

**Affiliations:** 1 Obstetrics and Gynecology Resident, Universidad del Rosario, Cll 24 # 29-95, Bogota, Colombia; 2 Obstetrics and Gynecology Department. Urogynecology and Pelvic Reconstructive Surgery, Hospital Universitario Mayor Mederi, Bogota, Colombia

**Keywords:** LeFort colpocleisis, Narrative review, Postoperative complication, Pyometra

## Abstract

**Introduction:**

LeFort colpocleisis is a surgical alternative for women with pelvic organ prolapse (POP). Complications are rare and typically minor. The accumulation of purulence within the uterine cavity, or pyometra, after this procedure is exceptionally uncommon. To date, only a handful of cases have been reported. Hereby, we present a narrative review on the current knowledge regarding the diagnosis and treatment of this atypical complication.

**Methods:**

A literature search was conducted using five different databases to identify previously published articles on this subject. Relevant keywords included colpocleisis, LeFort colpocleisis, pyometra, and reoperation. Studies describing complications after colpocleisis with concurrent hysterectomy or colpocleisis of vaginal vault were excluded.

**Results:**

Six case reports were identified, all from English-language sources. Data form an additional case at our institution was included. Median age was 78 years, and most women had multiple comorbidities. All underwent LeFort colpocleisis, with varying additional procedures. Only one case had a successful conservative approach; hysterectomy via laparotomy was ultimately required in the remaining cases. No cases of underlying malignancy were reported.

**Conclusions:**

Pyometra is a rare but important differential diagnosis in patients with a history of LeFort colpocleisis presenting with fever, vaginal discharge, and abdominopelvic pain. A high index of clinical suspicion is paramount, since prompt diagnosis and appropriate surgical management are a standard of care.

## Introduction

Pelvic organ prolapse (POP) is a common condition that increases with age. It is defined as the descent of one or more of the following anatomic structures: the anterior vaginal wall, the posterior vaginal wall, the uterus (cervix) or the vaginal vault [1]. Although there are different therapeutic options, surgery remains the main pilar of treatment and can be classified in two approaches: reconstructive surgery (RS) and obliterative surgery (OS). While the goal of RS is to restore the anatomical function and position of pelvic organs [2], in OS the vaginal cavity is not preserved making it a shorter and less invasive approach. OS is a good alternative for women with multiple medical comorbidities, for those who refuse or fail pessary treatment and for those uninterested in having penetrative vaginal intercourse [3–5]. Additionally, OS is considered a safer alternative than RS given the lower risk of perioperative complications, lower incidence of severe complications and lower incidence of urinary tract infection (UTI) rendered in some studies [6–8].

Obliterative procedures can be classified as follows: colpocleisis without hysterectomy (LeFort colpocleisis), colpocleisis with hysterectomy, and colpocleisis of vaginal vault. To accomplish obliteration, the vaginal epithelium on the anterior and posterior vaginal walls is removed and then the fibromuscular layers of both walls are sutured together [9]. When performing a colpocleisis without hysterectomy, epithelium-lined tunnels must be created bilaterally. These tunnels allow for identification of postmenopausal bleeding and serve as drainage for uterine secretions, including postoperative inflammatory exudate [3, 10].

OS is an effective and safe procedure: recurrence rates range from 0 to 9.3% and perioperative complications rates range from 4.6 to 15.2%. Complications are usually minor, and the majority of them occur postoperatively [2, 11–15]. Pyometra, the formation of an abscess inside the uterus [16], may develop after LeFort colpocleisis. However, this complication is rarely described in the medical literature.

The aim of this narrative review is to summarize reports in the literature of colpocleisis-related pyometra cases, including a case that occurred at our hospital. We additionally discuss risk factors, diagnosis, management, and prevention of this rare complication.

## Materials and Methods

The initial search was conducted between February and March 2025 across five databases: PubMed, SciELO, Cochrane, Embase, and Google Scholar. The analysis included publications from January 1990 through March 2025.

The following keywords were used for the literature search: colpocleisis, LeFort colpocleisis, colpectomy, vaginectomy, vaginal obliteration, pyometra, and reoperation. Of the above, only pyometra and reoperation correspond to Medical Subject Headings (MeSH). The following search strategy was used: (((colpocleisis) OR (LeFort colpocleisis) OR (colpectomy) OR (vaginectomy) OR (vaginal obliteration)) AND ((pyometra) OR (reoperation))). The literature search was conducted in both English and Spanish.

No results were found in Cochrane or SciELO. The search yielded 117, 18, and 8 titles in Google Scholar, Embase, and PubMed, respectively. The titles and abstracts of these publications were reviewed by all the authors. Articles related to the occurrence of pyometra following LeFort colpocleisis were included. Articles reporting complications after colpocleisis with hysterectomy or colpocleisis of vaginal vault were excluded. The search did not yield any narrative or systematic reviews.

## Results

Six case reports were identified [5, 10, 17–20]. All articles were published in English. One case occurred in Turkey and the remaining five in the United States (USA). A table was created on the basis of the information from the reviewed articles (Table 1). Data from an additional case that occurred at our hospital was included. General demographic and the available health related information (age, comorbidities, risk factors, results of preoperative exams), the initial surgical intervention, clinical presentation at the time of the postoperative complication, results of initial studies, and definitive treatment were included. Huseyin reported a case of pyometra rupture and septic shock following LeFort colpocleisis, but this case was not included due to the unavailability of the full text [21].

**Table 1:** Results and reviewed articles

Title	Author, publication year and country of origin	Demographic information	Initial intervention and clinical presentation	Initial complementary studies	Definitive treatment
Pyometra Following Le Fort Colpocleisis [10]	Kohli et al., 1996, USA	92 years old History of coronary disease and HTN Preoperative endometrial biopsy: benign	LeFort colpocleisis and Kelly plication of the bladder neck New prolapse after 8 months and second colpocleisis was performed Pelvic pain and lower limb edema 4 weeks later	WBC 21.800/μL Pelvic US: enlarged uterus with complex echogenic intracavitary mass	IV antibiotics (unspecified) + exploratory laparotomy, subtotal hysterectomy, intravaginal and intra-abdominal drain insertion
Pyometra and recurrent prolapse after Le Fort colpocleisis [17]	Roth 2007, USA	78 years old History of chronic obstructive pulmonary disease and tobacco abuse	LeFort colpocleisis and Kelly plication of the bladder neck New prolapse and purulent vaginal discharge for 3 years, unresponsive to oral antibiotics	Normal WBC (unspecified) Pelvic US: enlarged uterus with a complex echogenic intracavitary mass measuring 9 × 6 × 6 cm	Exploratory laparotomy, total hysterectomy, BSO, upper vaginectomy, Halban’s culdoplasty + new total colpocleisis with high levator plication and cystoscopy
Pyometra necessitating hysterectomy after colpocleisis in an extremely elderly patient [5]	Carberry et al., 2007, USA	95 years old History of HTN, senile dementia, arthritis and osteoporosis	Failed initial conservative management with pessary Dilation and curettage, LeFort colpocleisis, enterocele repair and cystoscopy – with administration of preoperative antibiotics Fever and foul-smelling vaginal discharge 4 weeks later	WBC 25.300/μL CT: enlarged uterus with fluid filling the endometrial cavity	IV antibiotics (unspecified) + vaginal drainage of pyometra Three days later: clinical deterioration → exploratory laparotomy, total hysterectomy and BSO Postoperative course was complicated by bilateral pleural effusion and lower limb DVT Final pathology: intrauterine and cervical abscess
Pyometra After Le Fort Colpocleisis Resolved With Interventional Radiology Drainage [18]	Shayya et al., 2009, USA	78 years old History of DM, HTN and morbid obesity Preoperative cervical cytology test and endometrial biopsy: benign	LeFort colpocleisis – Cefazolin was given as prophylactic antibiotic Fever, chills and rigors 14 days later	WBC 41,700/μL Sediment rate 104 mm/h CT: enlarged uterus with a fluid and gas collection	IV antibiotics (Piperacillin-Tazobactam and Vancomycin) + transabdominal drain insertion through the myometrium into the endometrial canal (by interventional radiology), with aspiration of 400 mL of purulent fluid (positive for Morganella Morganii) 72 h later → vaginal dilation of lateral tracts, irrigation with 3 L of normal saline with 1,000 units of Bacitracin and 80 mg of Gentamicin
Pyometra complicating a LeFort colpocleisis [19]	Toglia et al., 2009, USA	81 years old History of DM, HTN, breast cancer, angina, anemia and prior venous thromboembolism	LeFort colpocleisis Foul-smelling vaginal discharge and abdominal pain 4 weeks later, unresponsive to oral antibiotics	WBC 10,000/μL CT: fluid-filled endometrial cavity	IV antibiotics (Piperacillin-Tazobactam and Metronidazole) + exploratory laparotomy, subtotal hysterectomy and BSO Final pathology: pyometra
Management of pyometra after LeFort colpocleisis resistant to drainage [20]	Yasa et al., 2016, Turkey	72 years old Preoperative cervical cytology test: benign Preoperative pelvic US: thin endometrium	LeFort colpocleisis Fever and pelvic mass 55 days later	WBC 25,000/μL CRP 150 mg/dL CT: enlarged uterus filled with heterogeneous collection	IV antibiotics (Piperacillin-Tazobactam) + transabdominal drain insertion (by interventional radiology), draining 850 mL of purulent fluid (positive for E. Coli) Drain removed after completing 3 days without fever Recurrence 10 days later → exploratory laparotomy, total hysterectomy, BSO and upper vaginectomy
Pyometra – an Atypical Complication Following LeFort Colpocleisis: Narrative Review	Chala Saad et al., 2025, Colombia	69 years old History of DM, HTN and coronary disease Preoperative cervical cytology test: benign	LeFort colpocleisis with perineorrhaphy and transobturator mid-urethral sling – Cefazolin was given as prophylactic antibiotic Fever and urinary symptoms 3 days later	WBC 15,950/μL CRP 28.82 mg/dL CT: endometrial cavity distended with fluid and gas	IV antibiotics (Cefepime and Metronidazole) No clinical improvement after 10 days → exploratory laparotomy, subtotal hysterectomy and BSO Final pathology: extensive liquefactive necrosis of the endometrium, associated with abscess formation and ulceration

*BSO* bilateral salpingo-oophorectomy, *CRP* C-reactive protein, *CT* computed tomography, *DM* Diabetes mellitus, *DVT* Deep vein thrombosis, *HTN* Hypertension, *IV* Intravenous, *US* Ultrasound, *WBC* White blood count

The median age was 78 years (interquartile range 72–92). In one case there was no record of the patient’s medical history [20]. In the remaining cases, all patients had multiple comorbidities (diabetes mellitus (DM) and hypertension (HTN) were the most common). All patients underwent LeFort colpocleisis. As additional procedures, Kelly plication of the bladder was performed in two patients [10, 17], curettage + enterocele repair + cystoscopy was done in one patient [5] and, in our case, a transobturator mid-urethral sling was placed.

The use of antibiotics was not reported by Roth [17]. In the remaining cases (including ours) intravenous (IV) antibiotics were administered as part of the therapeutic regimen; only three cases (including ours) specified the use of prophylactic preoperative antibiotics [5, 18]. In three cases, immediate surgical management via hysterectomy by laparotomy was performed [10, 17, 19]. In the other three cases (including ours), initial conservative treatment failed and hysterectomy via laparotomy was ultimately required [5, 20]. Only one case had a successful conservative approach, with transabdominal intrauterine drainage along with vaginal dilation and irrigation of the lateral tracts using diluted antibiotics [18].

Only three cases (including ours) reported the final histopathology results, which confirmed the diagnosis of pyometra [5, 19]. No cases of malignancy were described, either based on intraoperative findings or histopathological evaluation.

## Discussion

In general, we found that pyometra as a complication of LeFort colpocleisis occurs in older women with multiple comorbidities. In the majority of cases, additional surgical procedures were performed and IV antibiotics were administered as part of the treatment. In six out of seven cases a hysterectomy was performed, either as immediate surgical treatment or due to failure of a conservative approach.

### Etiology

The etiology of pyometra following a colpocleisis without hysterectomy remains unclear. The first case was published in 1996 by Kohli [10] who proposed this complication results from obliteration of the lateral tunnels, thereby preventing adequate drainage of uterine secretions. In this case, the patient did not present with vaginal discharge.

However, Roth [17] challenged this theory by arguing that tunnel patency may not necessarily be compromised, as there would otherwise be no drainage of purulence into the vagina. In his case, the patient had a 3-year history of vaginal discharge that began shortly after the initial intervention. Hence, he proposed a different pathophysiological mechanism involving a temporary tunnel occlusion (allowing for an ascending infection to reach the uterine cavity) followed by spontaneous recanalization.

In the patient presented by Carberry [5], dilation and curettage were performed at the same surgical time of the LeFort colpocleisis. The pyometra was attributed to instrumentation of the uterine cavity. However, it is paramount to clarify that routine curettage or any other endometrial evaluation is not recommended in women with low-risk of endometrial cancer [22].

In the present case, none of the aforementioned theories fully explain the development of pyometra: the tunnels were patent as evidenced by the presence of vaginal discharge, disease progression time was not consistent with Roth’s theory and there was no intrauterine instrumentation. These observations compel us to consider pyometra as a multifactorial entity with different pathogenic mechanisms. The most likely risk factors for infection in this population include age (being that all the cases occurred in women above the age of 65) and the presence of comorbidities that can be risk factors for infection (diabetes, immunocompromised state, chronic illness).

### Diagnostic Approach

The clinical presentation of pyometra following an OS is variable. Roth presented a case of recurrent POP and chronic vaginal discharge lasting 3 years. In the remaining cases, symptoms and treatment occurred within the first 10 weeks after the surgical procedure.

In this review, the most common symptoms were fever and vaginal discharge. Abdominal and pelvic pain, chills, urinary symptoms, pelvic mass and lower limb edema were also reported. These findings are consistent with those from Ouh [16], who conducted a retrospective analysis of 65 women with pyometra not associated with colpocleisis. Vaginal discharge (73.9%), fever (16.9%), and lower abdominal pain (13.9%) were the most frequent symptoms.

When pyometra is clinically suspected, complementary diagnostic studies should be performed. Leukocytosis and elevated C-reactive protein (CRP) support the diagnosis [16]. However, patients undergoing colpocleisis are typically elderly women with multiple comorbidities, in whom the systemic inflammatory response may be blunted. Not all patients in the reviewed cases had fever or leukocytosis.

**Fig. 1:**
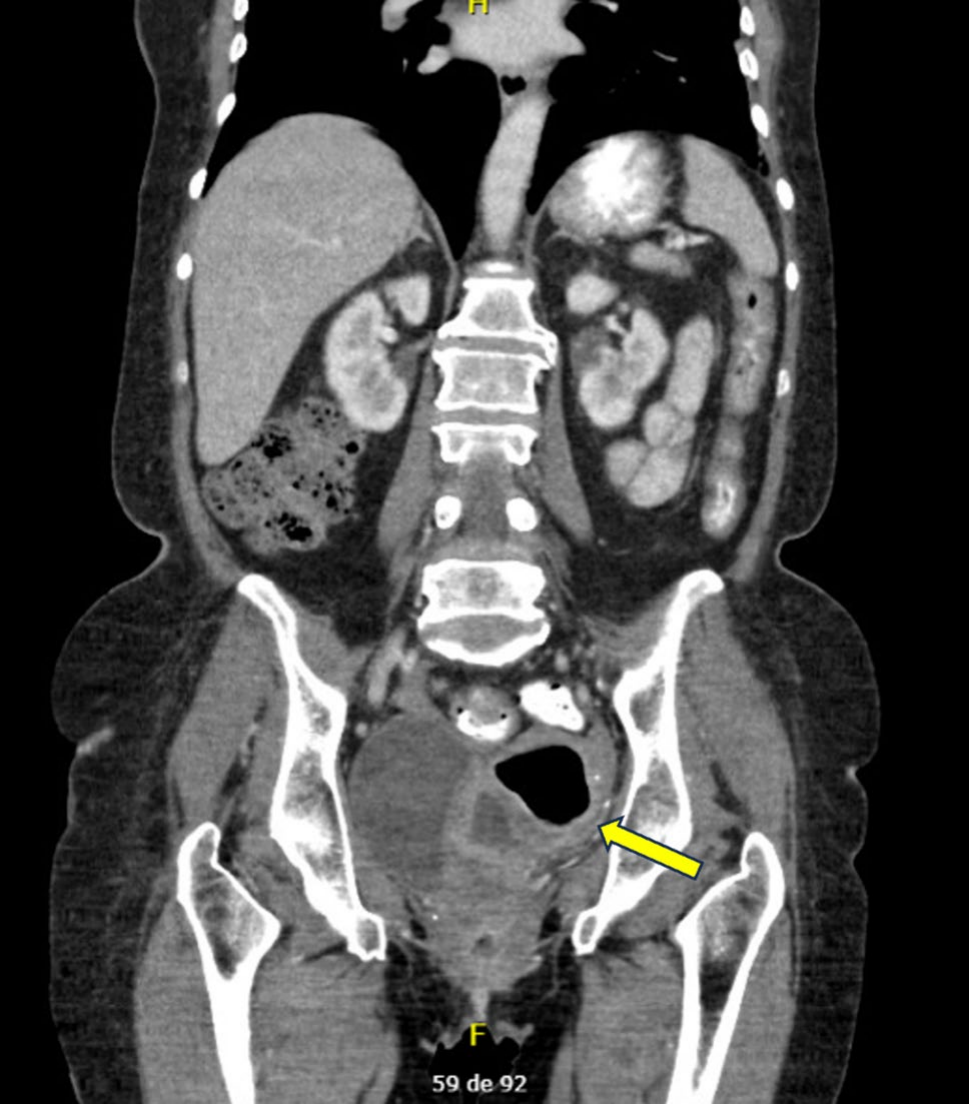
Coronal CT scan showing an air-fluid level within the uterine cavity

**Fig. 2:**
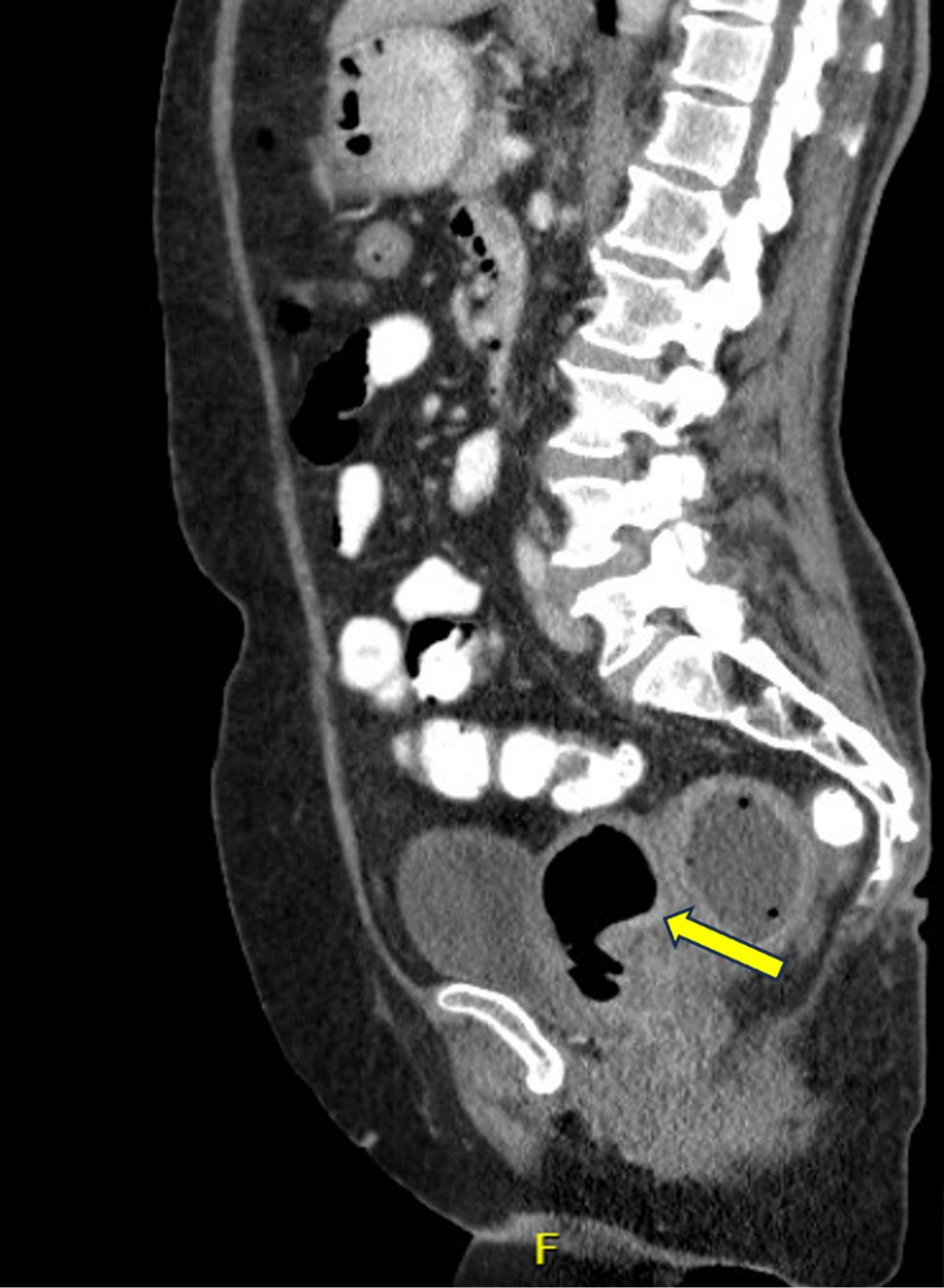
Sagittal CT scan demonstrating a retroflexed uterus with distension of the uterine fundus by gas and fluid

In light of the above, it is essential to perform imaging studies. Pelvic US is the first-line imaging modality. CT-scans or magnetic resonance imaging (MRI) may be considered for confirmation and evaluation of additional complications [23]. Characteristic findings include uterine enlargement, endometrial cavity distension, and heterogenous echogenic intrauterine content. CT and MRI can also identify the presence of intrauterine gas [20, 23]. The coronal and sagittal CT scans of our patient are presented in Figs. 1 and 2.

### Treatment

Antibiotics administration is the first pillar of treatment. However, given the scarcity of evidence on this subject, it remains unclear whether the microbiological profile of pyometra unrelated to colpocleisis is the same as that of cases associated with the procedure. Only two of the seven cases included in this review reported positive abscess and blood cultures for gram negative bacteria (see Table 1).

To guide management, we relied on data from studies on pyometra not associated with colpocleisis. Retrospective analyses by Ouh [16] and Jang [24] indicate that the most common pathogens are *Escherichia coli*, *Klebsiella pneumoniae*, and *Streptococcus spp*, followed by *Mycoplasma hominis* and *Ureaplasma species*. Therefore, empirical regimens should provide coverage for *Enterobacterales*, *Streptococcus* species and anaerobes.

The authors recommend the use of broad-spectrum antibiotics such as Piperacillin-Tazobactam or a fourth-generation cephalosporin plus Metronidazole. If a penicillin allergy is suspected, a fourth-generation cephalosporin may still be administered, as cross-reactivity between penicillin and cephalosporins is low ($\approx$ 2%) [25, 26]. If a true IgE-mediated allergy has been previously documented, the combination of Clindamycin and Gentamicin may be considered, although there is limited evidence supporting this recommendation [27].

The second pillar of treatment is a timely and appropriate source control. Both conservative and radical approaches were described in this review. Six out of seven patients ultimately required hysterectomy via laparotomy. In the only case of successful conservative management, drainage of the uterine cavity was performed through a transabdominal route, along with extensive irrigation of the lateral tunnels using diluted antibiotics. Taking this into account and considering the technical challenges of performing an effective transvaginal drainage of pyometra, we recommend hysterectomy as the preferred treatment for this complication. This allows immediate source control and prevents recurrence or systemic spread of the infection.

However, we acknowledge that there is insufficient evidence to give a strong recommendation regarding the best treatment approach. Hence, the placement of a transabdominal drain should be considered, particularly in elderly and/or frail patients.

### Prevention

Given the probable multifactorial nature of this condition, it is difficult to establish preventive measures other than performing a colpocleisis with concomitant hysterectomy. Nonetheless, it has been found that performing a hysterectomy at the same time increases perioperative morbidity [28–31]. In addition, the decision analysis model by Jones [32] suggests that colpocleisis with hysterectomy would only offer a protective benefit against unexpected malignant uterine pathology in women younger than 40 years of age. Thus, since LeFort colpocleisis is usually performed in women over the age of 70, the benefit of performing a routine concomitant hysterectomy is not clear.

The decision to perform colpocleisis with hysterectomy should be individualized, considering each patients’ comorbidities and risk factors for endometrial cancer [31], as well as their ability to tolerate a longer surgery, increased blood loss, the possibility of conversion to laparotomy, and bowel injury [33].

Evaluation of uterine bleeding and cervical pathology is challenging following LeFort colpocleisis [3]. We recommend preoperative endometrial assessment with a transvaginal pelvic US in patients with risk factors for endometrial cancer or with prior postmenopausal bleeding [3, 34]. Evidence suggests that in low-risk patients, performing no endometrial evaluation has a superior cost utility compared to routine transvaginal US and endometrial biopsy [22]. Hence, to avoid over screening, it is important to evaluate the individual risk of each patient, taking into account their medical history, risk factors, and previous symptomatology.

Regarding the appropriate control of comorbidities, it is well known that hyperglycemia in patients with diabetes is a risk factor for surgical site infection [35]. Thus, an adequate glycemic control prior to an OS is a standard of care. Other measures such as preoperative evaluation of possible sources of infection (bacterial vaginosis, UTI, history of constipation, etc.) may be considered, but there is insufficient evidence to give general recommendations.

### Additional Considerations

The main limitations of this study include the narrative nature of the literature review and the limited number of reported cases, given it is an uncommon complication. Furthermore, our case occurred at a referral center for urogynecologic pathology where the complexity of patients is typically higher than in the general population.

## Conclusions

Pyometra following LeFort colpocleisis is an atypical but serious complication. Despite its rarity, a high index of clinical suspicion is necessary in women presenting with vaginal discharge, fever, or abdominopelvic pain after the procedure. Treatment is mainly focused on intravenous antibiotics administration and prompt surgical source control.

## References

[1] Haylen BT, De Ridder D, Freeman RM, Swift SE, Berghmans B, Lee J, et al. An international urogynecological association (IUGA)/international continence society (ICS) joint report on the terminology for female pelvic floor dysfunction. Int Urogynecol J. 2010;21:5–26. 19937315 10.1007/s00192-009-0976-9

[2] Liang L, Ao S, Wang S, Chen Z, Peng L, Chen L, et al. Efficacy and safety of Le Fort colpocleisis in the treatment of stage III-IV pelvic organ prolapse. BMC Womens Health. 2024;24:618. 39567989 10.1186/s12905-024-03459-4 PMC11580651

[3] Karram MM, Hyde Y HT (2022) Obliterative procedures for pelvic organ prolapse. walters & karram urogynecology and reconstructive pelvic surgery. Fifth edition, Philadelphia: Elsevier; p 374–86.

[4] Cadena M, Dunivan G. Obliterative surgery for vaginal prolapse: an update. Curr Geriatr Rep. 2023;12:22–7.

[5] Carberry CL, Hampton BS, Aguilar VC. Pyometra necessitating hysterectomy after colpocleisis in an extremely elderly patient. Int Urogynecol J Pelvic Floor Dysfunct. 2007;18:1109–11. 17657546 10.1007/s00192-006-0285-5

[6] Sung VW, Weitzen S, Sokol ER, Rardin CR, Myers DL. Effect of patient age on increasing morbidity and mortality following urogynecologic surgery. Am J Obstet Gynecol. 2006;194:1411–7. 16647926 10.1016/j.ajog.2006.01.050

[7] Dessie SG, Shapiro A, Haviland MJ, Hacker MR, Elkadry EA. Obliterative versus reconstructive prolapse repair for women over 70-is there an optimal approach? Female Pelvic Med Reconstr Surg. 2017;23:23–6. 27782975 10.1097/SPV.0000000000000344 PMC5161604

[8] Mahon Coleman CE, Bonasia K, Pascali D, Clancy A. Complications of obliterative versus reconstructive vaginal surgery for pelvic organ prolapse in octogenarians: a retrospective cohort study. Neurourol Urodyn. 2024;43:1171–8. 38511609 10.1002/nau.25444

[9] Meriwether KV, Gold KP, De Tayrac R, Cichowski SB, Minassian VA, Cartwright R, et al. Joint report on terminology for surgical procedures to treat pelvic organ prolapse. Female Pelvic Med Reconstr Surg. 2020;26:173–201. 32079837 10.1097/SPV.0000000000000846

[10] Kohli N, Sze E, Karram M. Pyometra following Le fort colpocleisis. Int Urogynecol J Pelvic Floor Dysfunct. 1996;7:264–6. 9127184 10.1007/BF01901249

[11] Ugianskiene A, Glavind K. Follow-up of patients after colpectomy or Le Fort colpocleisis: single center experience. Eur J Obstet Gynecol Reprod Biol. 2021;262:142–6. 34022591 10.1016/j.ejogrb.2021.05.018

[12] Wang Y, Zhang K, Wang H, Yang J, Ying Y, Han J. Long-term efficacy and patient satisfaction of Le Fort colpocleisis for the treatment of severe pelvic organ prolapse. Int Urogynecol J. 2021;32:879–84. 32601781 10.1007/s00192-020-04380-8

[13] Mikos T, Chatzipanteli M, Grimbizis GF, Tarlatzis BC. Enlightening the mechanisms of POP recurrence after LeFort colpocleisis. Case report and review. Int Urogynecol J. 2016;28:971–8. 28025678 10.1007/s00192-016-3236-9

[14] Zebede S, Smith AL, Plowright LN, Hegde A, Aguilar VC, Willy Davila G. Obliterative LeFort colpocleisis in a large group of elderly women. Obstet Gynecol. 2013;121:279–84. 23344277 10.1097/AOG.0b013e31827d8fdb

[15] Espitia De La Hoz FJ. Colpocleisis: evolución y complicaciones en mujeres del Quindío, Colombia, 2009–2019. Rev Avances En Salud. 2020;4:12–23.

[16] Ouh YT, Oh DY, Hong H, Lee B, Kim C, Lee D, et al. Clinical characteristics of pyometra: eleven years of experience from a single institution. Clin Exp Obstet Gynecol. 2023;50:130.

[17] Roth TM. Pyometra and recurrent prolapse after le fort colpocleisis. Int Urogynecol J. 2007;18:687–8. 10.1007/s00192-006-0201-z 17001456

[18] Shayya RF, Weinstein MM, Lukacz ES. Pyometra after Le Fort colpocleisis resolved with interventional radiology drainage. Obstet Gynecol. 2009;113:566–8. 19155959 10.1097/AOG.0b013e3181952366

[19] Toglia MR, Fagan MJ. Pyometra complicating a lefort colpocleisis. Int Urogynecol J Pelvic Floor Dysfunct. 2009;20:361–2. 18773135 10.1007/s00192-008-0710-z

[20] Yasa C, Ugurlucan FG, Yalcin O. Management of pyometra after lefort colpocleisis resistant to drainage. Int Urogynecol J. 2016;27:645–6. 26768900 10.1007/s00192-015-2936-x

[21] Huseyin K, Tolga K, Kerem SD. Rupture of pyometra and septic shock after LeFort colpocleisis: a case report. Kuwait Med J. 2020;52:301–5.

[22] Kandadai P, Flynn M, Zweizig S, Patterson D. Cost-utility of routine endometrial evaluation before le fort colpocleisis. Female Pelvic Med Reconstr Surg. 2014;20:168–73. 24763159 10.1097/SPV.0000000000000043

[23] Zarour CC, Zaki-Metias KM, Kaur M, Mian A, Olson EB, Seedial SM. Idiopathic pyometra in a postmenopausal patient. Clin Imaging. 2021;80:145–7. 34329900 10.1016/j.clinimag.2021.07.002

[24] Jang S, Jeon M, Mun SJ, Kim SH. Clinical characteristics and risk factors for septic shock in patients with pyometra: a retrospective multicenter cohort study. J Infect Public Health. 2024;17:862–7. 38554592 10.1016/j.jiph.2024.03.019

[25] Shenoy ES, Macy E, Rowe T, Blumenthal KG. Evaluation and management of penicillin allergy: a review. JAMA. 2019;321:188–99. 30644987 10.1001/jama.2018.19283

[26] Picard M, Robitaille G, Karam F, Daigle JM, Bédard F, Biron É, et al. Cross-reactivity to cephalosporins and carbapenems in penicillin-allergic patients: two systematic reviews and meta-analyses. J Allergy Clin Immunol Pract. 2019;7:2722-2738.e5. 31170539 10.1016/j.jaip.2019.05.038

[27] Vanderah TW. Katzung’s basic & clinical pharmacology. Sixteenth edition. McGraw Hill. 2024.

[28] Von Pechmann WS, Mutone M, Fyffe J, Hale DS. Total colpocleisis with high levator plication for the treatment of advanced pelvic organ prolapse. Am J Obstet Gynecol. 2003;189:121–6. 12861149 10.1067/mob.2003.546

[29] Hill AJ, Walters MD, Unger CA. Perioperative adverse events associated with colpocleisis for uterovaginal and posthysterectomy vaginal vault prolapse. Am J Obstet Gynecol. 2016;214:501.e1-501.e6. 26529371 10.1016/j.ajog.2015.10.921

[30] Bochenska K, Leader-Cramer A, Mueller M, Davé B, Alverdy A, Kenton K. Perioperative complications following colpocleisis with and without concomitant vaginal hysterectomy. Int Urogynecol J. 2017;28:1671–5. 28470415 10.1007/s00192-017-3340-5

[31] Raina J, Bastrash MP, Suarthana E, Larouche M. Perioperative complication rates of colpocleisis performed with or without concomitant hysterectomy: a large population-based study. Int Urogynecol J. 2023;34:1111–8. 36705729 10.1007/s00192-023-05457-w PMC9881524

[32] Jones KA, Zhuo Y, Solak S, Harmanli O. Hysterectomy at the time of colpocleisis: a decision analysis. Int Urogynecol J. 2016;27:805–10. 26658894 10.1007/s00192-015-2903-6

[33] Buchsbaum GM, Lee TG. Vaginal obliterative procedures for pelvic organ prolapse: a systematic review. Obstet Gynecol Surv. 2017;72(3):175–83. 28304415 10.1097/OGX.0000000000000406

[34] Elkattah R, Brooks A, Huffaker RK. Gynecologic malignancies Post-LeFort Colpocleisis. Case Rep Obstet Gynecol. 2014;2014:1–5. 10.1155/2014/846745 PMC4266759 25525536

[35] Steiner HL, Strand EA. Surgical-site infection in gynecologic surgery: pathophysiology and prevention. Am J Obstet Gynecol. 2017;217:121–8. 28209490 10.1016/j.ajog.2017.02.014

